# Ovarian Mature Cystic Teratoma: Challenges of Surgical Management

**DOI:** 10.1155/2016/2390178

**Published:** 2016-03-24

**Authors:** Abha Sinha, Ayman A. A. Ewies

**Affiliations:** ^1^Sandwell and West Birmingham NHS Trust, Birmingham City Hospital, Birmingham B18 7QH, UK; ^2^The College of Medical & Dental Sciences, University of Birmingham, Birmingham, UK

## Abstract

Although ovarian mature cystic teratomas are the commonest adnexal masses occurring in premenopausal women, there are many challenges faced by gynecologists on deciding upon the best surgical management. There is uncertainty, lack of consensus, and variation in surgical practices. This paper critically analyzes various surgical approaches and techniques used to treat these cysts in an attempt to outline a unified guidance. MEDLINE and EMBASE databases were searched in January 2015 with no date limit using the key words “ovarian teratoma” and “ovarian dermoid.” The search was limited to articles in English language, humans, and female. The two authors conducted the search independently. The laparoscopic approach is generally considered to be the gold standard for the management. Oophorectomy should be the standard operation except in younger women with a single small cyst. The risk of chemical peritonitis after contents spillage is extremely rare and can certainly be overcome with thorough peritoneal lavage using warmed fluid. There is a place for surveillance in some selected cases.

## 1. Introduction

Adnexal masses are commonly encountered in gynecologic practice and often present both diagnostic and management dilemmas. In the United States, a woman has a 5–10% lifetime risk of undergoing surgery for a suspected ovarian mass or a cyst [1]. In premenopausal women, most adnexal masses are benign with an overall incidence of malignancy of only 1–3 : 1000 [2]. Ovarian mature cystic teratomas, also called dermoid cysts, are the most common germ cell tumor [3], accounting for up to 70% of benign ovarian masses in the reproductive years and 20% in postmenopausal women [4–6]. Immature cystic teratomas are rare (<3%) and usually occur in the postmenopausal age group [7]. In asymptomatic women, whether premenopausal or postmenopausal, with pelvic masses including ovarian mature cystic teratoma, transvaginal ultrasound scan (TVS) is the imaging modality of choice. No alternative imaging modality has demonstrated sufficient superiority to TVS to justify its routine use [1]. Ovarian mature cystic teratomas have a variety of appearances characterized by echogenic sebaceous material and calcification and typically contain a hypoechoic attenuating component with multiple small homogeneous interfaces, which were determined with 98% accuracy in a series of 155 cases [8].

There are controversies amongst gynecologists as regards the best surgical approach to manage ovarian mature cystic teratoma. There is paucity of well-designed comparative clinical trials to define the criteria as how to select a particular technique, and consequently there are variations in surgical practices. Various approaches and procedures were employed; however, laparoscopic approach has become the most popular and widely practiced in the past two decades. In this paper, we critically analyze various surgical approaches and techniques used to deal with ovarian mature cystic teratoma. Further, we suggest guidance for management of these lesions based on the best available evidence to help both women and gynecologists make suitable individualized decisions.

## 2. Controversies of Surgical Management

There are several differences in practice as regards surgical management of ovarian mature cystic teratoma. These relate to the following:

1. Surgical approach: laparoscopy versus laparotomy.
2. The procedure: oophorectomy versus cystectomy.
3. The relevance of the spillage of the contents and techniques to minimize it.
4. The place of ovarian tissue sparing techniques.
5. Specimen exteriorization: laparoscopic port versus colpotomy.
6. The criteria for surveillance [expectant management].
7. Management of torsion.

### 2.1. Surgical Approach: Laparoscopy versus Laparotomy

There is a consensus amongst the investigators that operative laparoscopy is the method of choice for removing ovarian mature cystic teratoma as it offers the advantages of less intraoperative blood loss, reduced postoperative pain, shorter hospital stay, fewer postoperative adhesions, and better cosmetic result. In the last two decades, two randomized controlled trials (RCT) including 42 laparoscopic operations versus 42 laparotomy operations [4, 9] and nine retrospective comparative studies including 655 laparoscopic operations versus 409 laparotomy operations for ovarian mature cystic teratoma highlighted the superiority of the laparoscopic approach over laparotomy [10–18]. A systematic review of six RCT compared the laparoscopic approach with laparotomy in a total of 324 women undergoing removal of ovarian cysts of various natures. Laparoscopy was associated with reduced febrile morbidity, postoperative pain, postoperative complications, overall cost, and earlier discharge from hospital [19].

Nonetheless, the laparoscopic approach was significantly associated with longer operating time [14, 17] and higher contents spillage rate [14, 16]. It was reported that contents spillage occurred in one-third of the laparoscopic cases and it was particularly associated with larger cysts and also in those cases treated with cystectomy [14]. Laberge and Levesque found that laparoscopic approach was significantly associated with longer operating time, higher rate of contents spillage (18% versus 1%), and higher rate of recurrence rate after laparoscopic ovarian cystectomy (7.6% versus 0%) when compared with laparotomy [17].

The guidelines of The Royal College of Obstetricians and Gynecologists (RCOG) in the UK recommend that when surgery is indicated, a laparoscopic approach be generally considered to be the gold standard for the management of all benign ovarian masses. Laparoscopic management is also cost-effective because of the associated earlier discharge from hospital and return to work. In the presence of large masses with solid components such as large mature cystic teratoma laparotomy may be appropriate. The maximum cyst size above which laparotomy should be considered is controversial [2]. Some investigators recommended laparotomy for mature cystic teratoma >10 cm [12].

In addition, the RCOG recommends that a surgeon with suitable experience and appropriate equipment should undertake laparoscopic management of benign ovarian cysts. The appropriate route for the surgical management of ovarian masses depends on several factors related to the woman (including suitability for laparoscopy and her wishes), the mass (size, complexity, and likely nature), and the setting (including surgeon's skills and equipment). A decision should be made after careful clinical assessment and counselling considering the above factors. Where appropriately trained staff and equipment are unavailable, consideration should be given to referral to another provider [2].

### 2.2. The Procedure: Oophorectomy versus Cystectomy

There is no data in the literature as regards the best procedure. Howard, in a case series (8 laparoscopic versus 12 laparotomy), documented that fertility status influenced the choice of cystectomy or oophorectomy as the surgical procedure for ovarian mature cystic teratoma [18]. A RCT (20 laparoscopic versus 20 laparotomy), including premenopausal women with ovarian mature cystic teratoma ≤10 cm treated with cystectomy, reported no recurrence in both arms after five years [20]. However, Laberge and Levesque, in their retrospective comparative study, reported significantly higher rates of contents spillage (18% versus 1%) and recurrence (7.6% versus 0%) during laparoscopic cystectomy (*n* = 95) when compared with laparotomy (*n* = 150). There was no associated morbidity with spillage [17].

In a case series of 56 women with ovarian mature cystic teratoma treated laparoscopically (with 48 undergoing cystectomy), Chapron et al. found no case of chemical peritonitis and two women developed recurrence [21]. Another noncomparative study, including 99 women with ovarian mature cystic teratoma treated by ovarian cystectomy through laparotomy, observed women for 5 years. Of the 99 women, 18 had bilateral mature cystic teratoma and 10 had multiple teratomas in a single ovary. Two women developed malignant germ cell neoplasms, and three developed a recurrent mature cystic teratoma in an ovary from which a teratoma was previously removed. Bilateral or multiple mature cystic teratomas were present at the initial operation in four out of these five women. The authors suggested that women with bilateral or multiple ovarian mature cystic teratoma may be at higher risk of recurrence and may have a greater tendency to develop future ovarian germ cell neoplasms [22].

The RCOG recommends that the possibility of removing an ovary should be discussed with the woman preoperatively. This discussion should be in the context of oophorectomy being either an expected or unexpected part of the procedure. The pros and cons of electively removing an ovary should be discussed, taking into consideration the woman's preference and the specific clinical scenario [2]. Ovarian cystectomy may be the technique of choice in younger women unless the patient chooses oophorectomy. Oophorectomy should be the standard operation in postmenopausal women and in perimenopausal women with multiple cysts in the same ovary or with large ovarian mature cystic teratoma where there is not much ovarian tissue to conserve [18, 22].

### 2.3. The Relevance of the Spillage of the Contents and Techniques to Minimize It

Chemical peritonitis, in case of spillage of ovarian mature cystic teratoma contents, is rare but difficult to treat. It was reported in different series to occur in <0.2% of cases [1]. The spillage of contents is significantly higher in laparoscopic approach when compared with laparotomy. The risk is highest when laparoscopic cystectomy is performed [11, 14–17, 21–24] with some studies reporting rates of 100% [25]. Nevertheless, there is a consensus amongst investigators that spillage does not lead to short or long term complications such as severe chemical peritonitis or persistent pelvic pain if a liberal peritoneal lavage is carried out at the end of the procedure with skimming of the floating debris with suction tube until clear [16, 17, 21, 25–28]. In fact, it could be argued that cyst contents spillage is easier and more efficiently treated when it occurs during laparoscopy rather than laparotomy because of the better exposure of the Pouch of Douglas and the feasibility of thorough peritoneal lavage to ensure minimal residues from spillage. In Albini et al. comparative study (19 laparoscopic versus 19 laparotomy), there was no chemical peritonitis or persistent pelvic pain after 11-month follow-up in the laparoscopy group despite having higher spillage rate [16]. Similar findings were also stated in two recent retrospective studies including 152 laparoscopic versus 107 laparotomy operations. Despite reporting >50% cyst content spillage rate associated with laparoscopic management, no case of chemical peritonitis was reported [29, 30].

Various techniques were used to reduce the spillage rate and the potential consequent chemical peritonitis. Three reports recommended the routine intraoperative use of an endoscopic retrieval bag. The authors either operated in the bag or exteriorized the sac intact in the bag [21, 23, 27]. Morelli et al., in a RCT discussed below, found that the mesial-side incision technique was associated with significantly less spillage of the cyst contents [3%] when compared with antimesial incision (20%) [31]. Kruschinski et al. recommended the gasless lift-laparoscopic approach which combines laparoscopy with the standard procedures of laparotomy and thus may help reduce the spillage of ovarian mature cystic teratoma contents. In 79 cases undergoing cystectomy, there were only three cases of cyst rupture but it was possible to avoid spillage by closing the lesion with a clamp and continuing the enucleation of the teratoma during a lift-laparoscopic operation. This technique involves using a reusable abdominal wall retraction system that allows the laparoscopic viewing of the abdomen to be combined with operation methods using conventional instruments. Raising the abdominal wall mechanically creates the working space required for the laparoscopic operation and flexible valve-free trocars are used allowing the introduction of both conventional and laparoscopic instruments simultaneously [24]. This technique is not very popular in Europe and Western Hemisphere but is a possible option in resource poor settings.

RCOG recommends that spillage of cyst contents should be avoided where possible as preoperative and intraoperative assessment cannot absolutely preclude malignancy. Consideration should be given to the use of a tissue bag to avoid peritoneal spill of cystic contents. If inadvertent spillage does occur, meticulous peritoneal lavage of the peritoneal cavity should be performed using large amounts of warmed fluid. Use of cold irrigation fluid may not only cause hypothermia, but will also make retrieval of the contents more challenging by solidifying the fat-rich contents. Any solid content should be removed using an appropriate bag [2].

### 2.4. The Place of Ovarian Tissue-Preserving Techniques

Some investigators described techniques to maximize ovarian tissues preservation during ovarian cystectomy for mature cystic teratoma. Zupi et al. proposed laparoscopic removal by combining hydrodissection and blunt dissection which allows removal of intact teratoma with maximum functional tissue sparing even when the cyst seems to fill the ovary and no surrounding ovarian cortex can be seen on transvaginal ultrasound scanning (TVS). Within a year following surgery, TVS revealed that ovarian residual cortex surrounding the cyst was not visible in 24 ovaries, whereas in 56 ovaries residual tissue volume was greater than 3 cm^3^, suggestive of maximum tissue sparing [32]. However, the credibility of this study is questioned due to the fact that it included mainly small cysts with mean diameter of 5.5 cm [range 2.1–15.0 cm] and there was no control group to compare the outcome with the traditional “stripping technique.” Stripping has been criticized because it involves excessive removal of ovarian tissue with loss of follicles and increased contents spillage rate.

A RCT assessed the mesial-side ovarian incision for laparoscopic cystectomy as an ovarian tissue-preserving technique. The mesial side is the anterior hilar margin of the ovary where the tubal fimbriae are closely applied to the tubal pole of the ovary. Women were randomized into either mesial incision group [*n* = 33, mean cyst size = 75 mm] or antimesial incision [*n* = 34, mean cyst size = 81 mm]. Women in both groups had similar characteristics in terms of age, cyst size, and basal hormone levels. The mesial-side incision technique was associated with significantly higher ovarian reserve in terms of lower FSH values and higher basal antral follicle number, ovarian diameter, and peak systolic velocity. In addition, the mesial-side incision technique was associated with significantly less contents spillage (3% versus 20%) probably because the ovarian cortex is thicker at the mesial side providing a better identification of cleavage plane. Moreover, this technique reduced operative time as there is faster identification of cleavage plane, easier enucleation, and less need for haemostasis [31]. With the standard laparoscopic cystectomy, Candiani et al. reported a 33% reduction in ovarian volume on TVS three months postoperatively. This was because of the loss of ovarian stromal tissue [33]. Li et al., in a prospective study of 191 women, also reported significant reduction in ovarian reserve reflected by the increase in mean FSH and decrease in antral follicle count probably due to damage secondary to electrocoagulation used in the standard laparoscopic cystectomy technique [34].

### 2.5. Specimen Exteriorization: Laparoscopic Port versus Colpotomy

Various exteriorization techniques were described in the literature for extraction of ovarian mature cystic teratoma following laparoscopy. Before the modern advances in laparoscopic surgery, colpotomy was used for specimen exteriorization with conflicting results. A prospective comparative study (*n* = 31) showed that this technique is significantly inferior to extraction through the port site in terms of operating time and intraoperative blood loss; however, a higher spillage rate (44% versus 19%) was observed in the laparoscopic port group when compared to colpotomy group [35]. On the other hand, another retrospective comparative study (*n* = 44) found that both cyst contents spillage (43% versus 79%) and operative time were significantly less when exteriorization occurred through colpotomy when compared with conventional laparoscopic techniques. Nonetheless, the mean estimated blood loss was higher in the colpotomy group (89 mL) when compared with laparoscopic port exteriorization (65 mL). Prophylactic antibiotics were used in colpotomy group since it is difficult to sterilize vaginal epithelium [36].

The standard technique for exteriorization is through the umbilical port. It involves placing the cyst after removal in a laparoscopic tissue retrieval bag, which is partially extracted through the umbilical incision. This was followed by suction irrigation and forceps removal of the contents until the collapsed cyst could be removed in the pouch [26].

The RCOG recommends that where possible removal of benign ovarian masses should be via the umbilical port. This results in less postoperative pain and a quicker retrieval time than when using lateral ports of the same size. Avoidance of extending accessory ports is beneficial in reducing postoperative pain, as well as reducing incidence of incisional hernia and incidence of epigastric vessel injury. It also leads to improved cosmesis [2].

### 2.6. The Criteria for Surveillance (Expectant Management)

Ovarian mature cystic teratomas grow over time, increasing the risk of pain and ovarian accidents. Therefore, surgical management is usually appropriate. There is no evidence-based consensus on the size above which surgical management should be considered. Most studies used an arbitrary maximum diameter of 5-6 cm amongst their inclusion criteria to offer expectant management [2, 37, 38]. The risk of torsion is higher in larger teratomas [39]. Caspi et al. prospective study, to evaluate the progress of ovarian mature cystic teratoma over 3–5 years, showed that the mean rate of growth of the cyst was 1.5–1.7 mm per year. The mean rate of growth was extremely slow in premenopausal women with initial cyst size of <6 cm. Therefore, they recommended surveillance with serial TVS for as long as the rate of growth is not more than 2 cm per year [38].

Although the TVS appearance of immature teratomas is nonspecific, the tumors are typically heterogeneous with scattered coarse calcifications and large irregular solid components. Surveillance is not recommended for such cases [40].

The American Congress of Obstetricians and Gynecologists (ACOG) guidelines stated that the frequency of repeat TVS or length of the follow-up has not been determined. The guidelines recommend repeat TVS when the morphology of the mass suggests benign disease and/or there is a compelling reason to avoid surgical intervention, for example, substantial risk for perioperative morbidity and mortality [1].

*Surveillance during Pregnancy*. A case series, including 127,177 deliveries over 13 years, found 63 (0.05%) cases of pelvic masses ≥5 cm. Antepartum surgery was performed in 17 patients (29%), 13 because of ultrasound findings suggestive of malignancy and 4 because of ovarian torsion. Only 4 women were found to have ovarian cancer or borderline malignancy. The remaining patients were observed, with surgery performed in the postpartum period or at time of cesarean delivery. The majority of masses (42%) were ovarian mature cystic teratomas [41].

The ACOG guidelines stated that adnexal masses diagnosed during pregnancy appear to have a very low risk for both malignancy and acute complications; therefore, they may be considered for expectant management. Surgical intervention may be indicated in pregnancy only in case of acute pain. Depending on gestational age, abdominal ultrasonography may be used in addition to TVS because the ovaries may be outside the pelvis later in gestation. Magnetic resonance imaging is the modality of choice if additional imaging is needed because it poses no fetal radiation exposure [1].

### 2.7. Management of Torsion

In most cases, ovarian mature cystic teratomas are asymptomatic with only 3-4% of women present with acute pelvic pain, which is usually due to torsion [14]. There is no role for expectant management in such cases and they need emergency surgery [14]. The risk of torsion is highest in ovarian mature cystic teratoma because of the long pedicle, and nearly all torted cases were >5-6 cms in size [39]. Unfortunately, there is no clinical, biological, or radiological sign that may exclude the diagnosis of adnexal torsion. The presence of flow at color Doppler imaging does not allow exclusion of the diagnosis. An emergency laparoscopy is recommended for adnexal untwisting, except in postmenopausal women where oophorectomy is recommended. A persistent black color of the adnexa after untwisting is not an indication for systematic oophorectomy since a functional recovery is possible. Ovariopexy is not routinely recommended following adnexal untwisting [42].

Untwisting of an ischemic adnexal mass has recently been advocated for most cases of torted adnexa especially in pediatric and adolescent population with almost complete recovery of ovarian function. Usually, the affected ovary regains some or all of its vitality and function. Nevertheless, when the ovary is completely necrotic, it may form an abscess and this should be promptly dealt with surgically [43].

## 3. Conclusion

A brief guide to surgical management of ovarian mature cystic teratoma is presented as follows:

(1) The laparoscopic approach is considered to be the gold standard for the management [II-2, A]:
  (i) where appropriately trained staff and laparoscopic equipment are unavailable, then consideration should be given to referral to another provider [II-3, C];
  (ii) the maximum cyst size above which laparotomy should be considered is controversial [II-3, C].
(2) Oophorectomy should be the standard operation in postmenopausal women and in perimenopausal women with multiple cysts in the same ovary or with large teratoma where there is no much ovarian tissue to conserve [II-2, B]:
  (i) ovarian cystectomy may be the technique of choice in younger women [III, C];
  (ii) the possibility of removing an ovary should be discussed with the woman preoperatively [III, C].
(3) The spillage of cyst contents should be avoided where possible as preoperative and intraoperative assessment cannot absolutely preclude malignancy and to avoid the potential risk of chemical peritonitis [II-2, C]:
  (i) consideration should be given to the use of a tissue retrieval bag [I, A];
  (ii) if inadvertent spillage does occur, meticulous peritoneal lavage should be performed using large amounts of warmed fluid [I, A].
(4) The role of ovarian tissues preservation techniques during ovarian cystectomy is uncertain. The reported techniques include the following:
  (i) combining hydrodissection and blunt dissection rather than stripping [II-2, C];
  (ii) the mesial-side ovarian incision [II-A].
(5) The exteriorization of the teratoma should be via the umbilical port rather than using lateral ports of the same size [II-2, B]:
  (i) extending the port entry site should be resisted [II-2, B].
(6) There is no evidence-based consensus on the size above which surgical management should be considered. There may be a room for surveillance when there are the following:
  (i) the size is ≤5-6 cm [II-2, C];
  (ii) there is a compelling reason to avoid surgical intervention, for example, substantial risk of perioperative morbidity and mortality [II-2];
  (iii) there are no ultrasonographic features suggestive of malignancy [II-2, A].
 The frequency of repeating TVS or length of the follow-up has not been determined [II-2, B].
 During pregnancy, the risk for acute complications is very low and expectant management may be considered. Surgical intervention may be indicated only in case of acute pain [III, C].
(7) There is no role of surveillance when torsion is suspected and emergency surgery is indicated. Adnexal untwisting is an option in younger women since functional recovery is possible. Ovariopexy is not routinely recommended following untwisting [II-2, B].

The laparoscopic approach is generally considered to be the gold standard for the management except for very large cysts. Oophorectomy should be the standard operation in postmenopausal women and in perimenopausal women with multiple cysts in the same ovary or with large teratoma where there is no much ovarian tissue to conserve. The risk of chemical peritonitis after contents spillage is extremely rare and can certainly be overcome with thorough peritoneal lavage using warmed fluid. The virtue of ovarian tissue-preserving techniques described in the literature is not established in credible studies. Exteriorization of the specimens should be through the umbilical port where possible. There may be a room for surveillance when the size is ≤5-6 cm, particularly in surgically high-risk women, but there is no data in the literature about the frequency or length of follow-up. In case of torsion, untwisting is an option especially in pediatric and adolescent population since a functional recovery is possible.
